# Electrospun Shape-Stabilized Phase Change Materials Based on Photo-Crosslinked Polyethylene Oxide

**DOI:** 10.3390/polym13172979

**Published:** 2021-09-02

**Authors:** Giulia Fredi, Parnian Kianfar, Sara Dalle Vacche, Alessandro Pegoretti, Alessandra Vitale

**Affiliations:** 1 Department of Industrial Engineering, University of Trento, 38123 Trento, Italy; alessandro.pegoretti@unitn.it; 2 INSTM—University of Trento Research Unit, 50121 Firenze, Italy; 3 Department of Applied Science and Technology, Politecnico di Torino, 10129 Torino, Italy; parnian.kianfar@polito.it (P.K.); sara.dallevacche@polito.it (S.D.V.); 4 INSTM—Politecnico di Torino Research Unit, 50121 Firenze, Italy

**Keywords:** phase change materials, electrospinning, thermal properties, shape stability, smart textiles

## Abstract

Phase change materials (PCMs) in the form of fibers or fibrous mats with exceptional thermal energy storage ability and tunable working temperature are of high interest to produce smart thermoregulating textiles, useful for increasing human thermal comfort while avoiding energy waste. Common organic PCMs suffer from instability in their molten state, which limits their applicability as highly performing fibrous systems. In this work, electrospun fibrous mats made of polyethylene oxide (PEO), a PCM with excellent thermal properties and biocompatibility, were fabricated and their shape instability in the molten state was improved through UV photo-crosslinking. The characterization aimed to assess the performance of these shape-stable electrospun mats as nanofibrous PCMs for thermal management applications. In addition to an enhanced resistance to water-based solvents, UV-cured electrospun PEO mats demonstrated a remarkable latent heat (≈112 J/g), maintained over 80 heating/cooling cycles across the phase change temperature. Moreover, their morphological stability above their melting point was demonstrated both macroscopically and microscopically, with the retention of the initial nanofibrous morphology. Tensile mechanical tests demonstrated that the UV crosslinking considerably enhanced the ultimate properties of the fibrous mat, with a five-fold increase in both the tensile strength (from 0.15 MPa to 0.74 MPa) and the strain at break (from 2.5% to 12.2%) compared to the uncrosslinked mat. In conclusion, the photo-crosslinked electrospun PEO material exhibited high thermal properties and good shape stability without displaying leakage; accordingly, in the proposed PCM system, the necessity for encapsulation or use of a supporting layer has been eliminated. Photo-crosslinking thus proved itself as an effective, fast, and environmentally friendly method to dramatically improve the shape-stability of nanofibrous PEO electrospun mats for smart thermoregulating textiles.

## 1. Introduction

Global warming and depletion of energy resources have become one of the most pressing issues of the century. These problems, exacerbated by the increasing industrialization and population growth, are now at the attention of the scientific and social communities and urgently claim for a prompt response. More remarkably, 13–15% of the global energy consumption comes from the thermal regulation of indoor areas, i.e., for HVAC (heating, ventilation, and air conditioning) [1,2]. This is even more impressive if we think that for an effective personal thermal regulation it would be sufficient to reach an optimal temperature range in the vicinity of the human body, which is instead sided by a considerable energy waste in heating and cooling the rest of the indoor space. Therefore, optimizing personal thermal management technologies would significantly reduce the energy consumption for HVAC, thereby favoring the transition towards a more sustainable indoor thermal management [1,2].

Considerable scientific and industrial effort has been put into developing personal heating/cooling systems, which mainly involve the production of smart thermoregulating textiles, able to enhance human thermo-physiological comfort [3,4]. In such textiles, thermal regulation can be active or passive, depending on whether or not an external energy source is needed to activate the heating/cooling mechanism [5]. Active technologies with an external hot/cold source or energy supply (e.g., liquid- or air-cooling garments) can provide more reliable and accurate body temperature control. However, besides depending on an external energy supply, they are commonly bulky and heavy and can hardly be applied in everyday life. Conversely, passive technologies rely on an external trigger such as a change in the level of temperature or moisture. They are generally more lightweight and portable and can fulfil their function without the need for an external energy supply.

Among the passive thermal management systems are those based on phase change materials (PCMs). PCMs are smart and functional materials that absorb a large amount of latent heat during phase transition and can then release it where and when it is needed, thereby filling the gap between thermal energy availability and demand. In fact, PCMs can store and retrieve energy through a liquid-gas, solid-liquid, or solid-solid transition over a narrow temperature range, in response to a temperature variation in the environment. An ideal PCM should meet crucial requirements such as a suitable transition temperature, large phase change enthalpy, proper thermal conductivity, cyclic durability, and shape stability during phase transition, as well as availability and low costs [6,7,8,9,10]. PCMs have been applied in solar energy storage [11], heat management in electronic devices [12], temperature-regulating textiles [13,14,15], smart building materials [16], biomedical field [17], and food packaging [18].

Organic solid-liquid PCMs, such as paraffin waxes, fatty acids, and polyethylene oxides (PEO), or polyethylene glycols, which can store a large amount of latent heat and have a working temperature that can be tuned by adjusting the molecular weight [11,19,20], are the most diffused PCM class, especially in the low-mid temperature range (0–100 °C). PEO in particular has gained significant momentum thanks to its high latent heat of fusion and chemical stability, but especially to its biocompatibility, water solubility, and non-toxicity. However, PEO suffers from shape instability in the molten state and needs to be confined to avoid leakage and loss of material above the melting temperature. One of the most effective and economically viable strategies to address this problem is the so-called “shape-stabilization”, achieved by embedding PEO in a polymer matrix with superior melting temperature [16,21], by encapsulating it in an organic or inorganic shell [22,23,24], by preparing composites and blends [25,26], and by using chemical methods such as grafting [27], crosslinking [28,29,30], and copolymerization [31]. These methods exploit different physical/chemical phenomena to avoid molten PEO leaking out of the system and the prepared PEO-based PCM losing its integrity.

Among the various applications of shape-stable PCMs, the fabrication of fibrous materials with phase change characteristics and temperature regulating properties has attracted great academic and industrial interest for promising applications in smart fabrics and thermoregulating clothes [32,33,34], packaging [35], biomedical applications [36], and heat storage/retrieval systems [37,38,39]. In these applications, shape stabilization has been reached through polymer blending, copolymerization, or the development of a core-sheath structure [33], which are always obtained with the help of a second polymer phase.

There are various techniques to incorporate PCMs into fibrous structures, such as melt/wet spinning, coating, lamination, and electrospinning [40]. Electrospinning is particularly interesting as it is a versatile technique used to produce ultrafine fibers, in the range of a few nanometers to micrometers. Electrospun phase change fibers feature high flexibility, controllable morphology, lightness, and specific high surface area, which promotes heat exchange and mitigates the low thermal conductivity of organic PCMs. To the best of the authors’ knowledge, PEO has never been utilized on its own to produce PCM electrospun nanofibers without a support layer or a containing sheath, and this is the challenge this work aims to tackle.

An interesting way to produce shape-stable PEO nanofibers is through chemical crosslinking, which results in the formation of covalent bonds between polymeric chains. When the reaction is induced by a light source, photo-crosslinking takes place, which is advantageous thanks to its low-energy consumption, fast conversion, precision, and eco-friendly characteristics [41,42,43]. Photo-crosslinking can be performed on PEO upon UV-irradiation; the addition of a proper photoinitiator and a suitable crosslinker can enhance the reaction efficiency. In fact, the photoinitiator abstracts hydrogen atoms from PEO chains, thus generating free radicals that can combine together, yielding a crosslinked network [44], while the crosslinker promotes the photo-crosslinking reaction at lower irradiation doses, thereby reducing scission of C-O bonds and polymer degradation [45]. If this process is performed onto an electrospun PEO membrane, the result is a crosslinked fibrous material that is water insoluble and resistant to solvents, as demonstrated in our previous works [43,46].

Herein, we demonstrate that, interestingly, such crosslinked PEO electrospun membranes macroscopically keep their shape also above the PEO’s melting temperature. The goal of the present study is thus to characterize PEO-based electrospun membranes as shape-stable PCMs, to assess their heat management properties and shape-stability, and to explore their applicability in the field of smart thermoregulating textiles. To reach this goal, electrospun PEO mats were produced and photo-crosslinked with the help of a photoinitiator and a multifunctional acrylic crosslinker. Their properties were compared to those of two reference samples, namely a PEO electrospun mat without the crosslinker and a PEO/crosslinker cast film. The characterization first aimed at assessing the crosslinking efficiency by Fourier Transform Infrared (FTIR) spectroscopy and gel fraction measurements, and the fibrous morphology resistance in presence of a water-based solvent. Then, a broad-ranging thermal characterization was performed to evaluate the performance of the PEO electrospun mat as a potential PCM, through the measurement of melting temperature and enthalpy and the evaluation of the fibrous morphological stability over several heating-cooling cycles. Finally, the mechanical and viscoelastic properties were evaluated through tensile tests and dynamic-mechanical thermal analysis (DMTA).

## 2. Materials and Methods

### 2.1. Materials

**Scheme 1:**
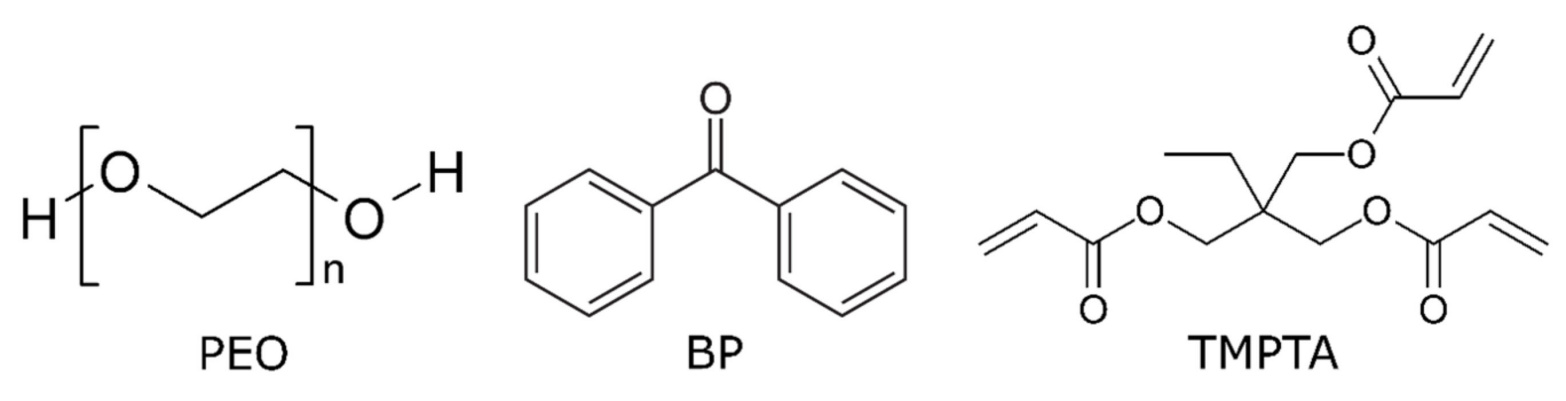
Chemical structures of the materials used in this work.

High molecular weight PEO (MW 1,000,000 g/mol), benzophenone (BP) (≥99%) as the photoinitiator, and trimethylolpropane triacrylate (TMPTA) (MW 296 g/mol) as the crosslinker were purchased from Merck KGaA (Darmstadt, Germany). The chemical structure of PEO, BP, and TMPTA is reported in Scheme 1.

### 2.2. Sample Preparation

**Scheme 2:**
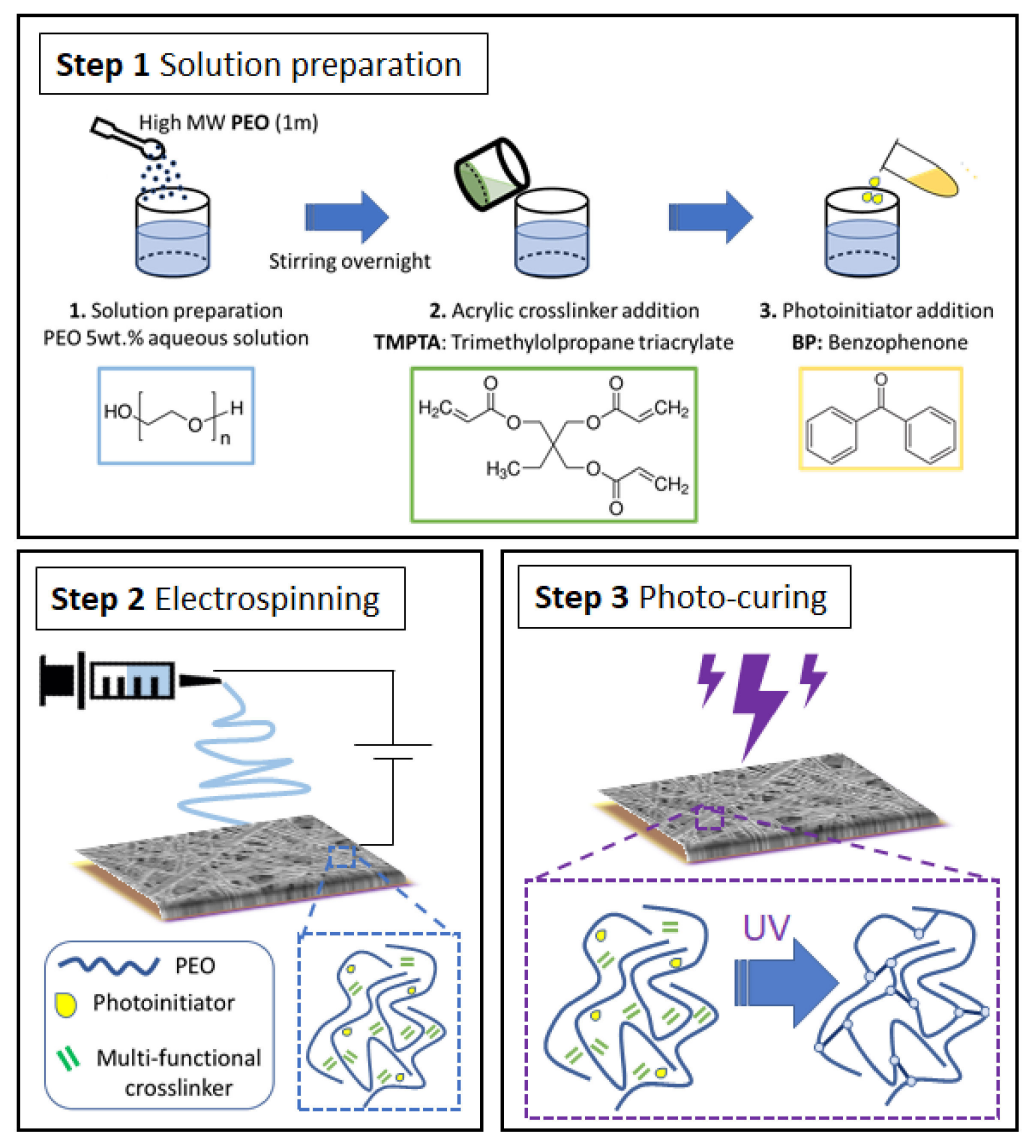
Schematic representation of PEO-based electrospun PCM fabrication procedure.

An electrospun PEO mat containing BP and TMPTA was produced and photo-crosslinked (sample named PEO-XL-E). Scheme 2 represents the fabrication procedure for such PEO-based electrospun PCM. The properties of PEO-XL-E were compared to those of two reference samples, namely an electrospun PEO mat without TMPTA (PEO-E) and a photo-crosslinked PEO/TMPTA cast film (PEO-XL-C). Table 1 lists the prepared samples with their labels, composition, and processing method. The details of sample preparation are reported hereafter.

#### 2.2.1. Electrospinning

The solution for the electrospinning of the PEO-XL-E sample was prepared by adding PEO to distilled water (concentration 5 wt.%) and magnetically stirring overnight at room temperature. Subsequently, TMPTA (PEO/TMPTA *w*/*w* 75/25) and BP (2 wt.% with respect to PEO) were added to the solution. The electrospinning solution for the sample PEO-E was prepared in the same way but without the addition of TMPTA.

Electrospinning was carried out by an E-fiber electrospinning system SKE apparatus (SKE Research Equipment^®^, Bollate, MI, Italy) equipped with a high voltage power supply, a syringe pump, a needle with a tip diameter of 1 mm, and a stationary collector covered by aluminum foils as the substrate. Electrospun mats with a thickness of few tens of microns were obtained at room temperature at a relative humidity of 45%, by applying a voltage of 8–12 kV and a flow rate of 0.1–0.2 mL/h, with a needle-to-collector distance of 15 cm.

#### 2.2.2. Film Casting

PEO-XL-C sample in the form of a film was produced starting from a solution with the same composition of PEO-XL-E sample. The solution was cast onto a glass Petri dish and water was left to evaporate for 48 h before testing.

#### 2.2.3. Photo-Crosslinking

All samples were subjected to UV light through a Dymax ECE 5000 UV-Curing flood lamp (Dymax Corporation, Torrington, CT, USA). The irradiation was carried out in the curing chamber under a constant flow of N_2_ as a purge gas to prevent the oxygen quenching phenomena. A UV light intensity of 70 mW/cm^2^ was applied on the sample surface for 20 min. The selection of UV dose (i.e., time and intensity of irradiation) is explained in our previous work [43].

### 2.3. Characterization

#### 2.3.1. Morphology

The morphology of the as-prepared electrospun mats was characterized via a ZEISS Supra 40 (ZEISS, Oberkochen, Germany) field-emission scanning electron microscope (FE-SEM). Before the experiment, samples were sputter-coated with a thin (~10 nm) layer of Cr film, deposited by a Quorum Q150T ES sputter coater (Quorum Technologies Ltd., East Sussex, UK). The size distribution of fibers and the full width at half maximum (FWHM) of its curve, and the surface porosity of the fibrous mats were calculated via ImageJ software and the DiameterJ plugin [47].

The morphology of the PEO-XL-E sample was also analyzed after contact with distilled water. For the PEO-E mat, a drop of water was placed on the sample and, after it dried, FE-SEM imaging was performed on the edge of the water drop.

#### 2.3.2. Photo-Crosslinking Efficiency

FTIR spectra were taken before and after UV irradiation using a Thermo Fisher Scientific Nicolet^TM^ iS50 Spectrometer (Thermo Fisher Scientific, Waltham, MA, USA) in attenuated total reflection (ATR) mode. All spectra were acquired in the range of 4000–500 cm^−1^ by 32 scans and a resolution of 4 cm^−1^. The measurement of the degree of conversion of the acrylate C=C bond of TMPTA was obtained by considering the area under the C=C band (approx. 1640 cm^−1^) of the reactive group ($A_{C=C}$) with respect to the area of a reference signal in the spectra, namely the C=O band at 1720 cm^−1^ ($A_{C=O}$), before irradiation (at time *t* = 0) and after UV-irradiation (at time *t*). Percent C=C conversion is calculated as indicated in Equation (1):

(1) Conversion %=1−AC=CAC=OtAC=CAC=Ot=0×100

The value of conversion for each sample was provided as an average value ± standard deviation of three repetitions.

The insoluble fraction of the samples PEO-XL-E and PEO-XL-C was evaluated through the gel content experiment. Samples were wrapped in a metallic mesh with ultrafine pores and soaked in distilled water, which is a suitable solvent for uncured PEO polymer as well as for TMPTA unreacted monomer. Samples were soaked for 24 h and subsequently were left drying in laboratory conditions for 48 h, and afterwards the mass loss of the samples was calculated.

#### 2.3.3. Mechanical and Dynamic-Mechanical Analysis

For the characterization of the mechanical properties, rectangular specimens with nominal in-plane dimensions of 30 × 4 mm^2^ were cut out of the prepared films or membranes with scissors and mounted on the instrument with a gauge length of 10 mm (calculated as the distance between the grips). The width of each specimen was measured with an optical microscope and the thickness with an electronic micrometer with an area of approx. 20 mm^2^.

Tensile tests were performed at room temperature with a TA DMA Q800 (TA Instruments, New Castle, DE, USA) equipped with a 16 N load cell. Five specimens were tested per sample. Each specimen was subjected to a pre-load of 0.005 N and a displacement ramp of 500 µm/min until fracture. The elastic modulus E was calculated as the maximum slope of the stress-strain curve. The test also allowed the determination of the ultimate tensile strength (UTS), corresponding to the maximum stress, and of the strain at break ($\varepsilon_{b}$).

DMTA was performed with a TA Instruments DMA Q800 (TA Instruments, New Castle, DE, USA) equipped with a 16 N load cell. Tests were performed in tensile mode between −80 and 100 °C, at 3 °C/min, with a strain amplitude of 0.05% applied at a frequency of 1 Hz.

#### 2.3.4. Heat Storage Properties and Long-Term Stability

Differential scanning calorimetry (DSC) was performed with a Mettler DSC 30 (Mettler Toledo, Inc., Columbus, OH, USA) between −60 °C and 100 °C at 10 °C/min, under a nitrogen flow of 100 mL/min. Specimens with a mass of approx. 5 mg were subjected to a first heating scan, a cooling scan, and a second heating scan. This test allowed the measurement of the melting and crystallization temperatures ($T_{m},\;T_{c}$) as the maximum of the endothermic peak in the heating scan and of the exothermic peak in the cooling scan, respectively, and melting and crystallization enthalpy values ($\Delta H_{m},\;\Delta H_{c}$) as integrals of the peaks. Moreover, to understand the long-term stability and thermal performance of the prepared samples, cyclic DSC tests were performed on the sample PEO-XL-E. A specimen was subjected to 50 heating-cooling cycles between 0 °C and 100 °C, at 10 °C/min. The test allowed the determination of the melting and crystallization enthalpy for each cycle.

Thermal cycling was also performed on a slightly bigger scale, with a climatic chamber DiscoveryMy DM340 C (Angelantoni Test Technologies Srl, Perugia, Italy). Specimens with a surface area of approx. 1 cm^2^ were subjected to 80 thermal cycles between 10 °C and 100 °C, at 3 °C/min. Samples were collected after 1, 20, and 80 cycles and subjected to FE-SEM analysis, with the same equipment and parameters described previously, to verify the morphological stability of the electrospun fibers.

## 3. Results and Discussion

PEO-based mats containing a multifunctional acrylic crosslinker (TMPTA) and benzophenone as photoinitiator were prepared by electrospinning (PEO-XL-E) and then photo-crosslinked by irradiation with UV light to enhance their physico-chemical properties, particularly their shape-stability characteristics both in the presence of solvents and elevated temperature. The formulation and the process conditions to prepare this sample were optimized in a previous work [38]. Herein, additionally, as reference samples, electrospun fibrous mats of PEO added only with photoinitiator (PEO-E), i.e., without the crosslinker, and PEO/crosslinker/photoinitiator continuous cast films (PEO-XL-C) were fabricated and UV-irradiated. A detailed description of the samples is in Table 1.

### 3.1. Crosslinking Efficiency and Fibrous Morphology

**Figure 1:**
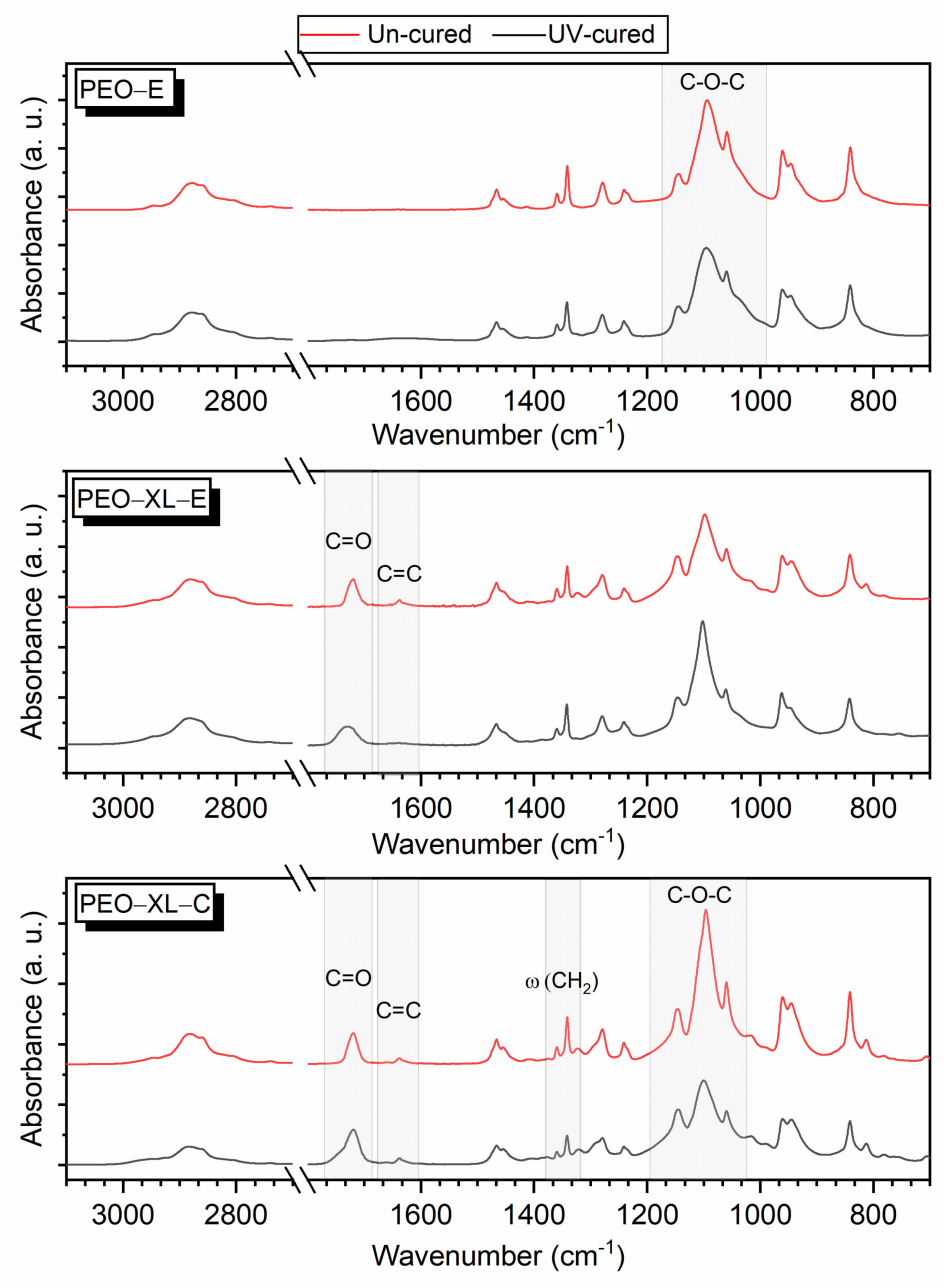
FTIR spectra of the PEO-E, PEO-XL-E, and PEO-XL-C samples before (red line) and after (black line) UV irradiation.

ATR-FTIR spectroscopy before and after UV-irradiation (Figure 1) was carried out to elucidate its effect on the chemical structure of the samples (PEO-E, PEO-XL-E, and PEO-XL-C). The characteristic peaks of neat PEO are found for all samples at 2876 cm^−1^ (methylene stretching), 1466 cm^−1^ (C-H bending), 1360 cm^−1^ and 1341 cm^−1^ (CH_2_ wagging), 1279 cm^−1^ (CH_2_ twisting), and 1145 cm^−1^, 1100 cm^−1^, and 1060 cm^−1^ (triplet peak due to -C-O-C- absorption complex). TMPTA, where present, is identified by absorption peaks at 1638 cm^−1^ (vinyl groups) and 1720 cm^−1^ (intense carbonyl peak).

It is well known that PEO can undergo chain scission and photo-oxidation, as well as photo-crosslinking, during UV irradiation; however, photo-oxidation can be excluded if such a process is performed in an inert atmosphere. Additionally, as shown in Figure 1, PEO-E does not exhibit photo-degradation (in terms of disappearance of characteristic peaks), and the slightly lower intensity and higher width of the ether bands in the area 1000–1180 cm^−1^ can indicate the occurrence of crosslinking phenomena [48].

For the systems containing the acrylic crosslinker either in the form of a fibrous mat (PEO-XL-E) or in the form of a film (PEO-XL-C), the photo-crosslinking reaction can be monitored by following the decrease of the C=C band with UV irradiation (Figure 1). For the electrospun PEO-XL-E sample, the peak intensity at 1638 cm^−1^ related to the acrylic reactive groups varies considerably upon UV irradiation, which indicates a certain degree of conversion (Table 2).

Similarly, the cast film PEO-XL-C shows a decrease in the intensity of C=C bands after UV irradiation. Additionally, this sample presents a decrease in the intensity of the triple ether bond (-C-O-C-) with a wider peak band, which indicates the PEO crosslinking in an inert atmosphere [48]. As this feature is not detectable in the PEO-XL-E sample, it can be supposed that the crosslinking efficiency is higher for the cast film compared to the electrospun mat. Moreover, the PEO-XL-C film after UV irradiation exhibits a lower intensity for the double peak with maxima at 1340 cm^−1^ and 1360 cm^−1^, which is a crystallinity marker in the PEO FTIR spectrum [48]. The irradiation thus decreases the crystallinity of PEO-XL-C, as confirmed by DSC measurements (described below). Finally, PEO photo-degradation cannot be detected either on the fibrous mat PEO-XL-E or on the continuous film PEO-XL-C.

After UV irradiation, the resulting C=C conversion is quite low for both PEO-XL-E and PEO-XL-C, being 45% and 38%, respectively (Table 2). As discussed in our previous work [43], the reason lies in the branched structure of trifunctional TMPTA, which causes some acrylic double bonds to be inaccessible due to their position in the polymer structure, and therefore to remain unreacted after UV irradiation. Nevertheless, the number of functional C=C groups that do react is sufficient to yield the successful crosslinking of the material, confirmed by gel content measurements. In fact, the crosslinked (insoluble) fraction was approx. 76% in PEO-XL-E and 96% in PEO-XL-C (Table 2). The lower crosslinking efficiency in the fibrous mat (as also evidenced by FTIR spectroscopy) could be due to its particular morphology: a higher surface area and the presence of pores can reduce the possibility for the monomer molecules to be in proximity of other reactive species and thus to react with each other.

**Figure 2:**
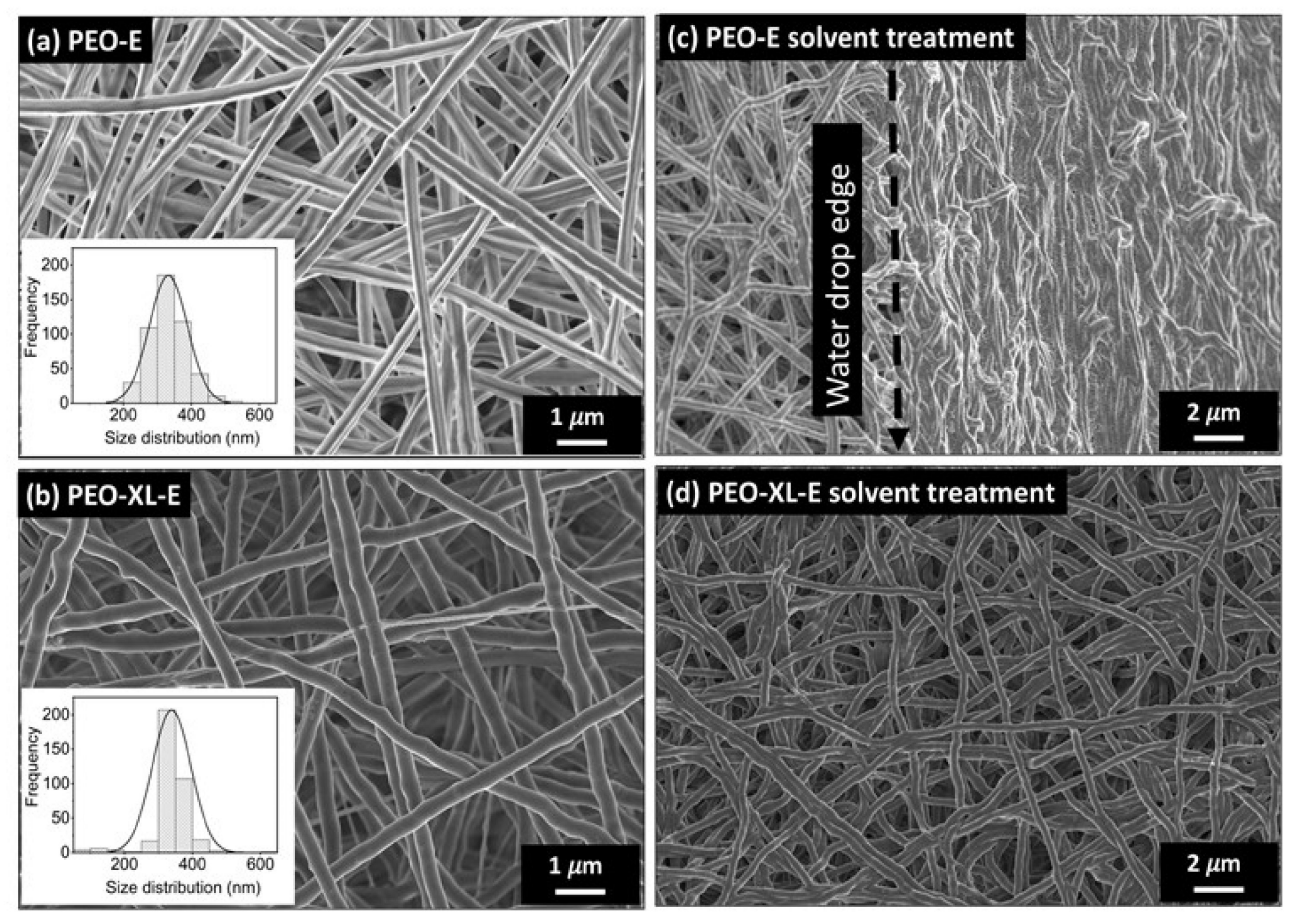
FE-SEM images of electrospun mat of PEO-E and PEO-XL-E fibers after UV irradiation (**a**,**b**) and after water treatment (**c**,**d**), respectively. The insets report the diameter distribution of fibers.

The FE-SEM micrographs of the fibrous PEO-E and PEO-XL-E mats after electrospinning and UV irradiation are shown in Figure 2a,b, respectively, together with the distribution of the fiber diameters. On average, the sample PEO-E was made of fibers with a diameter of 312 ± 40 nm and the sample PEO-XL-E of 356 ± 35 nm.

One of the aims of the crosslinking treatment is to make the fibrous mat resistant to solvents. In fact, an uncrosslinked PEO electrospun mat in contact with water first loses its fiber morphology and then solubilizes completely. To evaluate the retention of the fibrous morphology after contact with water, FE-SEM imaging was performed on the irradiated samples after water treatment. Figure 2c clearly shows that the sample PEO-E starts to lose fiber morphology under the effect of a water droplet (placed on the right side of the figure), while the sample PEO-XL-E retains its fibrous morphology (Figure 2d). Therefore, the photoinitiator alone does not allow the production of a solvent-resistant PEO-E mat due to the insufficient crosslinking, while the combination of photoinitiator and crosslinker successfully leads to the obtainment of a water-resistant and shape-stable fibrous PEO-based mat.

### 3.2. Mechanical and Dynamic-Mechanical Analysis

**Figure 3:**
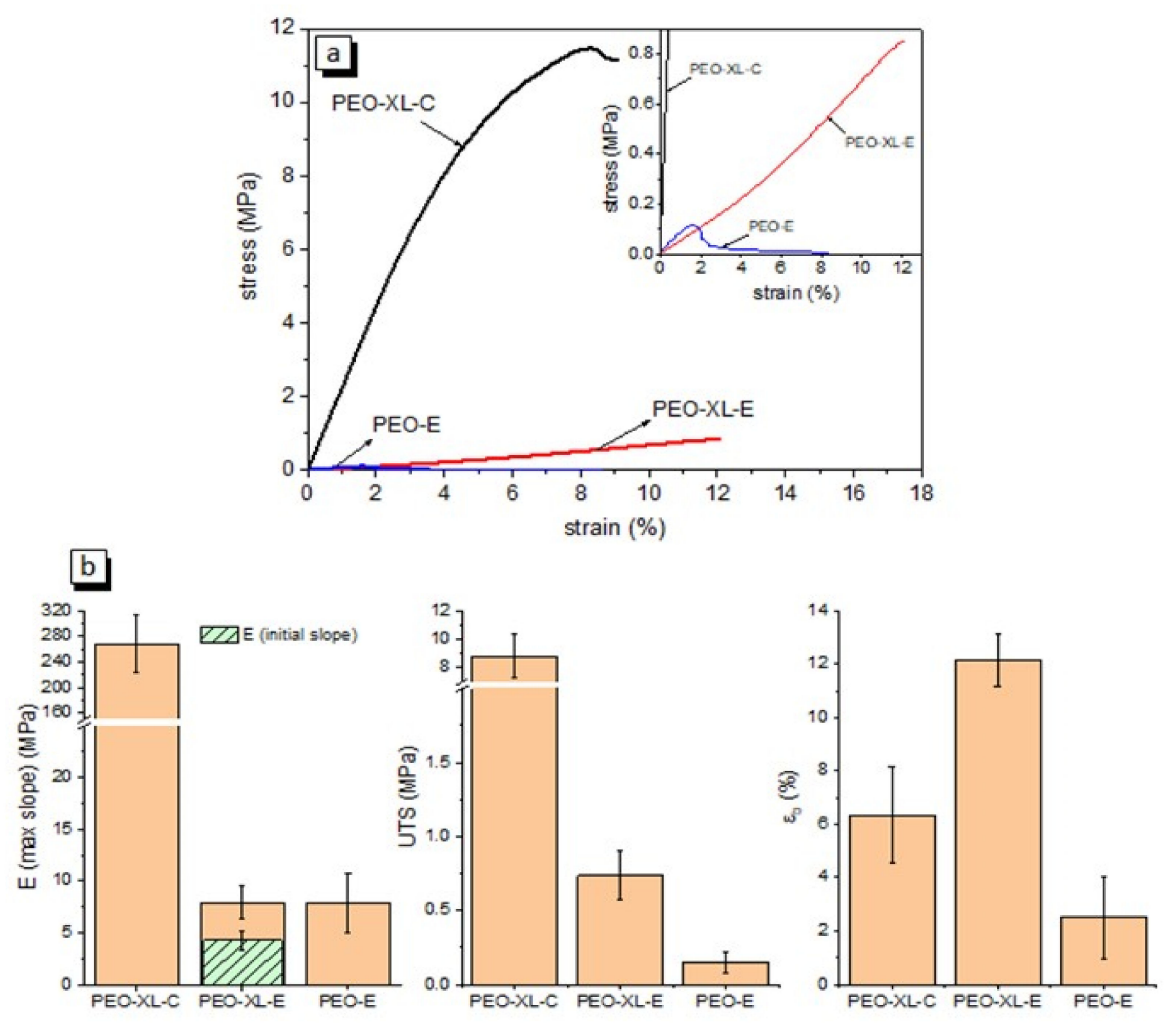
Results of quasi-static tensile tests. (**a**) Representative stress-strain curves; (**b**) elastic modulus (*E*), tensile strength (*UTS*), and strain at break (*ε_b_*) of the prepared samples (mean value and standard deviation).

Figure 3 shows the results of the quasi-static tensile tests. For the crosslinked fibrous mat (PEO-XL-E) the stress increases with strain until failure, and the initial slope is lower than that at higher strain values, as clearly observable from the inset in Figure 3a. This behavior is probably due to the gradual alignment of fibers parallel to the applied force alongside the presence of crosslinks. Additionally, for the photo-crosslinked film sample (PEO-XL-C), the stress increases with strain until failure, but the slope of the stress-strain curve decreases progressively with increasing strain. These two samples both undergo a catastrophic failure immediately after the maximum stress. Conversely, the sample PEO-E shows the opposite failure behavior: the stress increases with strain up to a maximum and then it decreases progressively, as the sample fails slowly by tearing.

The mechanical properties of the three samples are summarized in Figure 3b, in terms of elastic modulus (E), ultimate tensile strength (UTS), and strain at break ($\varepsilon_{b}$). Two values of E are reported for PEO-XL-E, the first calculated from the initial slope and the second from the maximum slope. The other samples show a single E value, since the initial and the maximum slopes always coincide. The film sample PEO-XL-C shows an elastic modulus of 268 ± 44 MPa and a mechanical strength of 8.8 ± 1.5 MPa, considerably higher than those of both fibrous membranes, as expected considering their porous morphology. By a comparison between the properties of the samples PEO-XL-E and PEO-E, it can be seen that the UV-crosslinking process considerably enhances the failure properties of the fibrous membrane, as the average value of UTS increases from 0.15 MPa to 0.74 MPa (+393%) and $\varepsilon_{b}$ from 2.5% to 12.2% (+388%).

**Figure 4:**
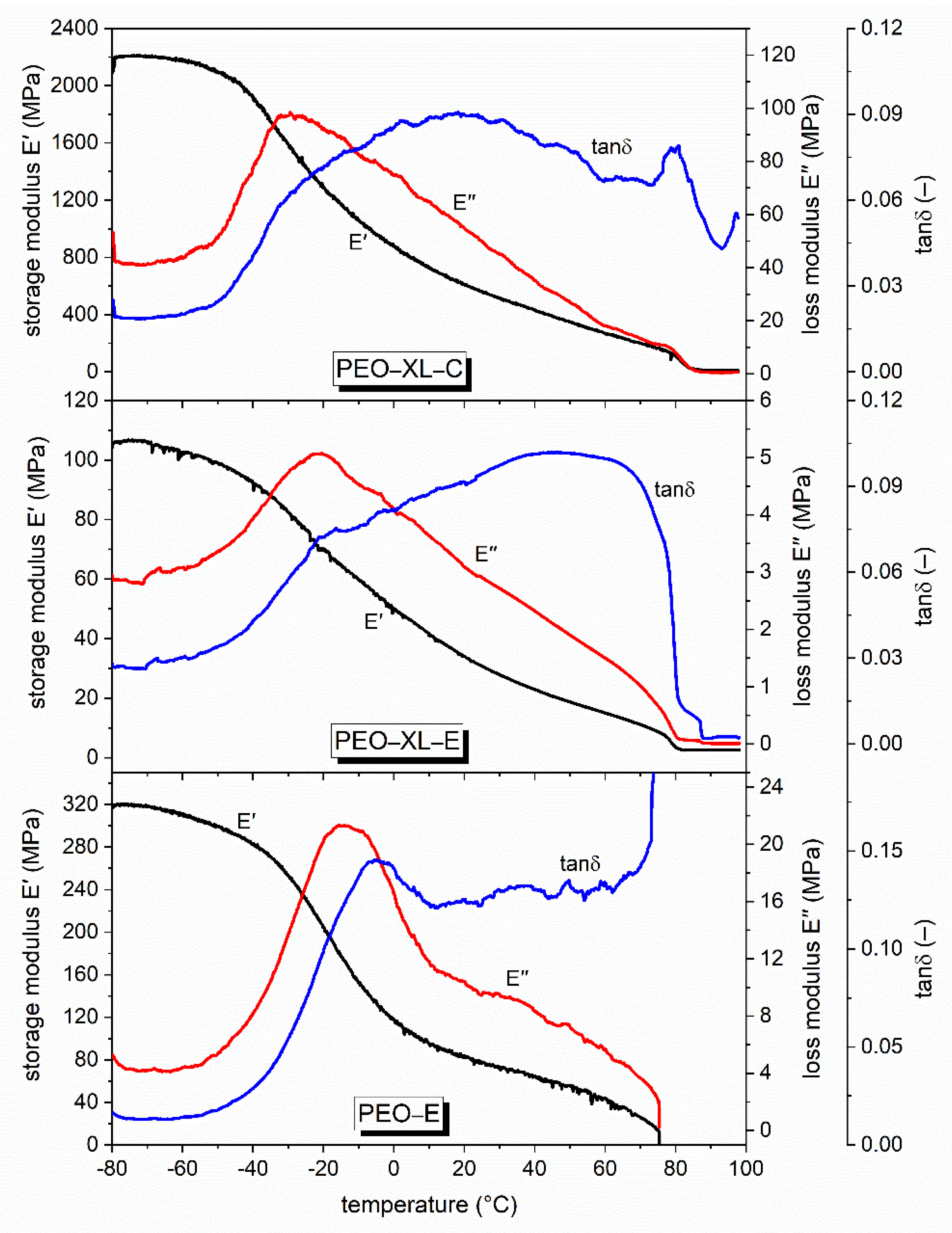
Results of DMTA tests. Storage modulus (*E′*), loss modulus (*E″*), and tanδ as a function of temperature. *E″* and tanδ have been smoothed.

The thermomechanical behavior of the prepared samples was further characterized through DMTA tests. Figure 4 shows the results of these tests, with trends of storage modulus ($E^{\prime }$), loss modulus (E″), and loss factor (tanδ) as a function of temperature. Tests were carried out starting from −80 °C up to 100 °C, to evaluate both the glass transition temperature of PEO (approx. −35 °C) and the mechanical stability of the samples above PEO’s melting temperature (approx. 80 °C).

For all samples, $E^{\prime }$ decreases with increasing temperature throughout the whole investigated temperature range. As expected, the fibrous membrane PEO-E melted at 78 °C and broke in two pieces during the measurement, while both the specimens UV-crosslinked by an acrylic crosslinker (PEO-XL-E and PEO-XL-C) maintained their integrity and could be tested until the end of the test, after which the samples could be retrieved from the testing grips as an integer piece. This evidences that an efficient UV-crosslinking is effective in stabilizing PEO not only against dissolution in water, but also against melting, and this is true for both the film and fibrous mat morphologies, thus suggesting their suitability as shape-stabilized phase change materials. However, in both cases, the values of $E^{\prime }$ above the melting point are very low, being approx. 14 MPa for PEO-XL-C and 1.3 MPa for PEO-XL-E at 90 °C, which correspond to a decrease of 97% and 96%, respectively, compared to the values at 20 °C.

It is important to notice that the crosslinking treatment does not suppress completely the melting transition of PEO. In fact, the melting signal is observable in both UV-crosslinked samples as a step in $E^{\prime }$ and E″ and, for PEO-XL-C, also as a small peak in tanδ. This signal is evident between 75 °C and 80 °C for PEO-XL-E and at a slightly higher temperature (between 80 °C and 85 °C) for PEO-XL-C. Interestingly, DMTA allowed monitoring the behavior of the PCM across the phase transition and investigating the impact of this transition on the viscoelastic parameters, which is an uncommon application for DMTA. Similar results have already been observed in previous works of our group [49,50,51,52,53] dealing with polymer-matrix composites containing an organic solid-liquid PCM. The present work demonstrated that this technique can be applied also to a shape-stabilized PCM by itself, which, to the best of the authors’ knowledge, has never been reported before in the open scientific literature.

DMTA results evidence an additional transition at lower temperatures, highlighted by a distinct peak in the signal of E″ (between −40 °C and 0 °C) and related to the glass transition of PEO. The $T_{g}\;$ values are reported in Table 3 as average ± standard deviation of at least two specimens per composition. To further investigate the effect of the irradiation on the glass transition of the systems, DMTA was performed on two additional samples, i.e., a neat PEO film (non-UV-irradiated) and a PEO film containing BP and UV-irradiated after casting, thus having the same composition and the same irradiation conditions of the sample PEO-E (see Figure S1 and Table S1 in the Supplementary Materials). The $E^{\prime \prime }$ peak temperature of the neat PEO film was found at −34 ± 1 °C, compatible with what is reported in the literature [54,55], while that of the UV-irradiated PEO/BP film was found at −22 ± 2 °C. Therefore, the non-irradiated system shows the lower $T_{g}$ value, which is increased when the samples are irradiated due to crosslinking phenomena, and all the UV-irradiated systems present a similar $T_{g}$, in the range from −28 to −17 °C. These results confirm that PEO could be slightly crosslinked after UV-irradiation in presence of photoinitiator solely [56]. However, it has to be noted that the acquired data of E″ are relatively noisy, and smoothing was applied to identify a peak temperature (see Figure S1 in the Supplementary Materials). This may have contributed to data uncertainty and increased the standard deviation. In any case, DMTA has proven a promising technique to assess the integrity and the viscoelastic properties of crosslinked samples below and especially above the melting temperature of PEO, which suggests that these samples can be suitably applied as shape-stabilized PCMs.

### 3.3. Heat Storage Properties and Long-Term Stability

**Figure 5:**
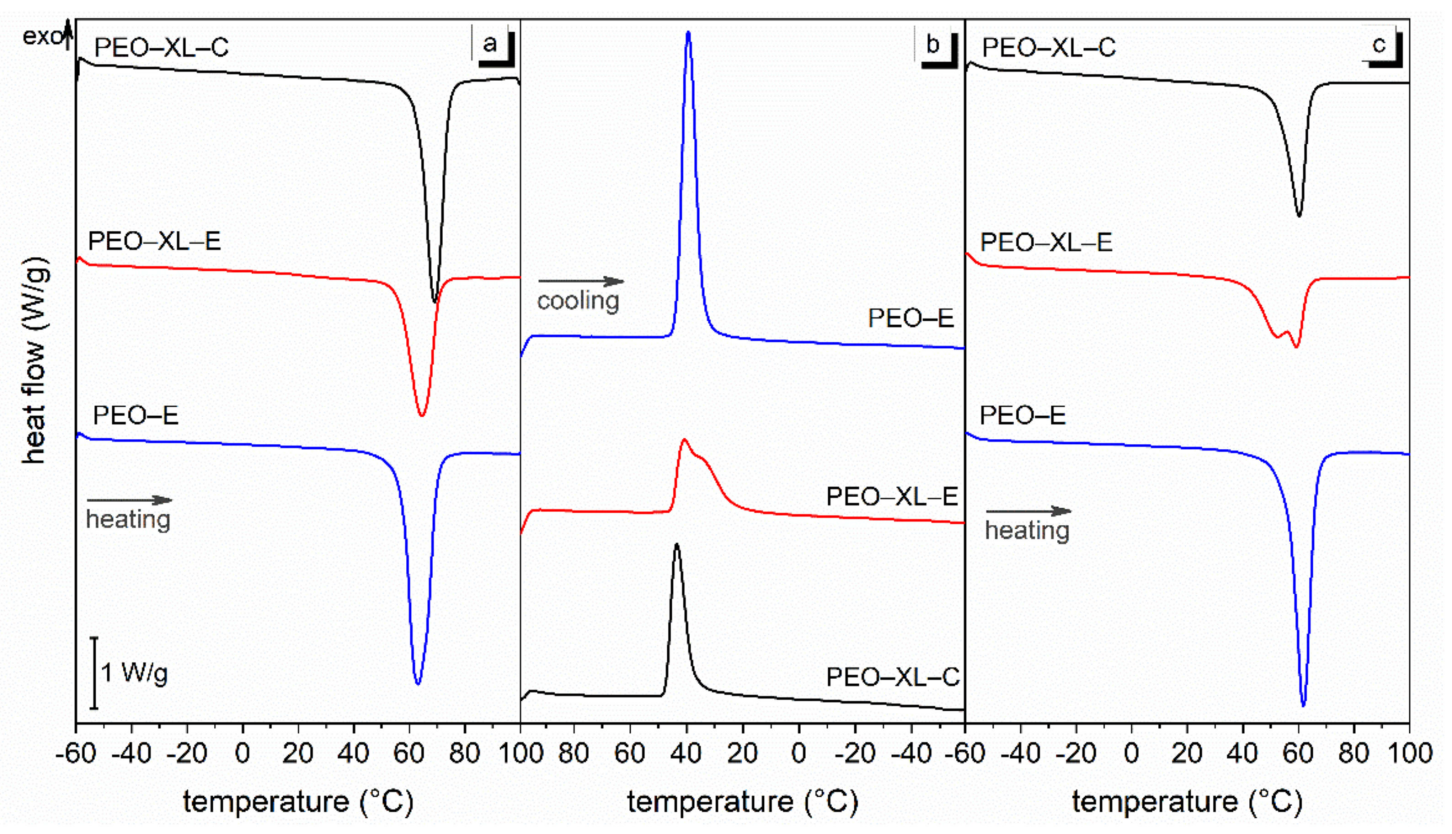
Results of DSC tests on the prepared samples. (**a**) first heating scan; (**b**) cooling scan; (**c**) second heating scan.

The heat storage and management properties and long-term thermal stability of the prepared samples have been investigated in small-scale experiments through DSC and in medium-scale experiments via a climatic chamber. Figure 5 and Table 4 show the results of DSC tests, performed as a heating-cooling-heating sequence between −60 °C and 100 °C. In the first heating scan, the uncrosslinked fibrous membrane PEO-E shows a single narrow melting peak at 62.2 °C, paralleled by a crystallization peak in the cooling scan and a second melting peak in the second heating scan. The melting enthalpy, as reported in Table 4, decreases from 186 J/g at the first scan to 170 J/g at the second, probably due to the different crystallization process occurring during electrospinning (when high drawing forces are applied) and during DSC analysis. For PEO-XL-C and PEO-XL-E samples, only the PEO chains can crystallize; the $\Delta H_{m}$ and $\Delta H_{c}$ values obtained from DSC measurements were thus normalized over the PEO weight only (i.e., 75 wt.% of the total weight of the sample). As expected, UV-crosslinking in the presence of TMPTA slightly decreases the melting enthalpy of the cured samples, especially in the second scan. Such a decreased ability to store and release heat can be due to the hindering of crystallization of the PEO chains when crosslinked [42]. However, the peak temperatures of melting and crystallization are unchanged, and thus they are not modified by the crosslinking process.

Unlike the other two samples, the fibrous membrane PEO-XL-E shows two local maxima in the crystallization peak and the subsequent melting peak; this is probably due to a different chain microstructure arrangement and inhomogeneous crosslinking.

**Figure 6:**
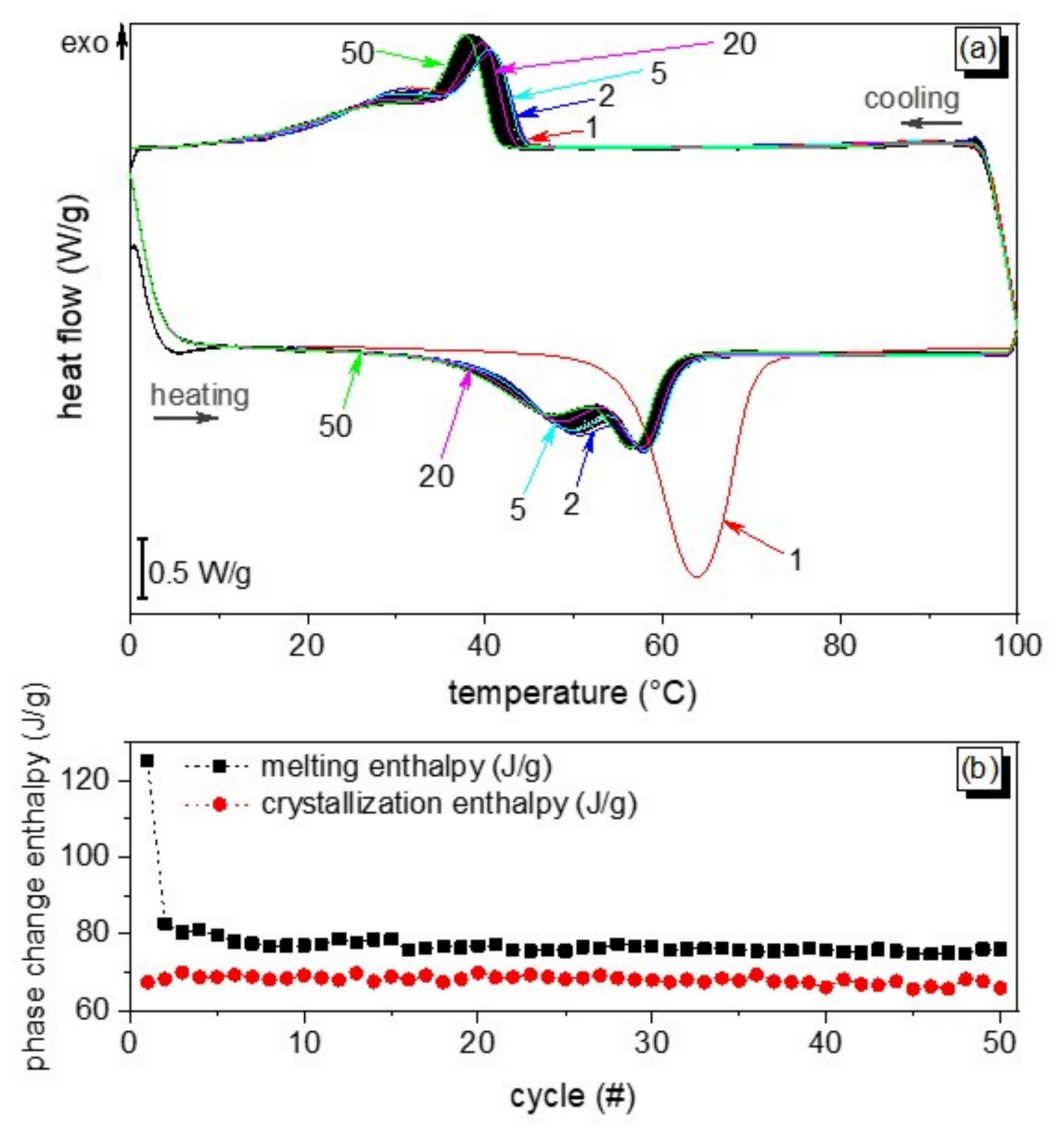
Results of cyclic DSC tests on the sample PEO-XL-E. (**a**) thermograms of cycles #1, #2, #5, #20 and #50 are colored and indicated with an arrow, while all the other cycles are black. (**b**) values of melting and crystallization enthalpy for all cycles.

In order to characterize the heat storage/release properties of the photo-crosslinked PEO-based fibrous mat (PEO-XL-E) after many thermal cycles, a cyclic DSC test was carried out in which the sample was thermally cycled 50 times. The results of this test are reported in Figure 6, which shows all 50 heating-cooling thermograms and the values of melting and crystallization enthalpy measure on each of them. While the first melting peak shows a single, well-defined minimum, the other peaks are very similar among each other and show two local maxima. As observed in single-scan DSC tests (Figure 5), the first melting peak has a temperature considerably higher than the others, while after the second cycle the enthalpy values stabilize. Therefore, although some enthalpy is lost between the first and the second cycle, this is definitely a first-cycle effect, after which the thermal storage properties of the fibrous membrane are stable over many subsequent cycles.

To further extend this characterization, the specimens subjected to the multiple DSC cycles were kept in laboratory conditions (23 °C, RH 40%) for 15 days and then retested with a heating-cooling cycle at 10 °C/min. The melting enthalpy measured in this test was 80.9 J/g, very close to that of cycles #2–#50, which suggests that, after the first cycle, the thermal properties of the material remain stable even after prolonged rest.

To collect more information on the temperature trends during thermal cycling and to assess the thermal performance and the thermal management properties of the samples on a larger scale than that allowed by DSC tests, additional samples of PEO-XL-E of approx. 5 cm^2^ were thermally cycled in a climatic chamber and some key temperature values were measured through thermocouples. As shown in Figure S2 (see Supplementary Materials for further information), a layer of PEO-XL-E helps to mitigate the temperature peaks. In fact, the maximum temperature difference between the chamber and the electrospun sample surface is higher than the temperature difference between the chamber and the aluminum foil used as a reference.

**Figure 7:**
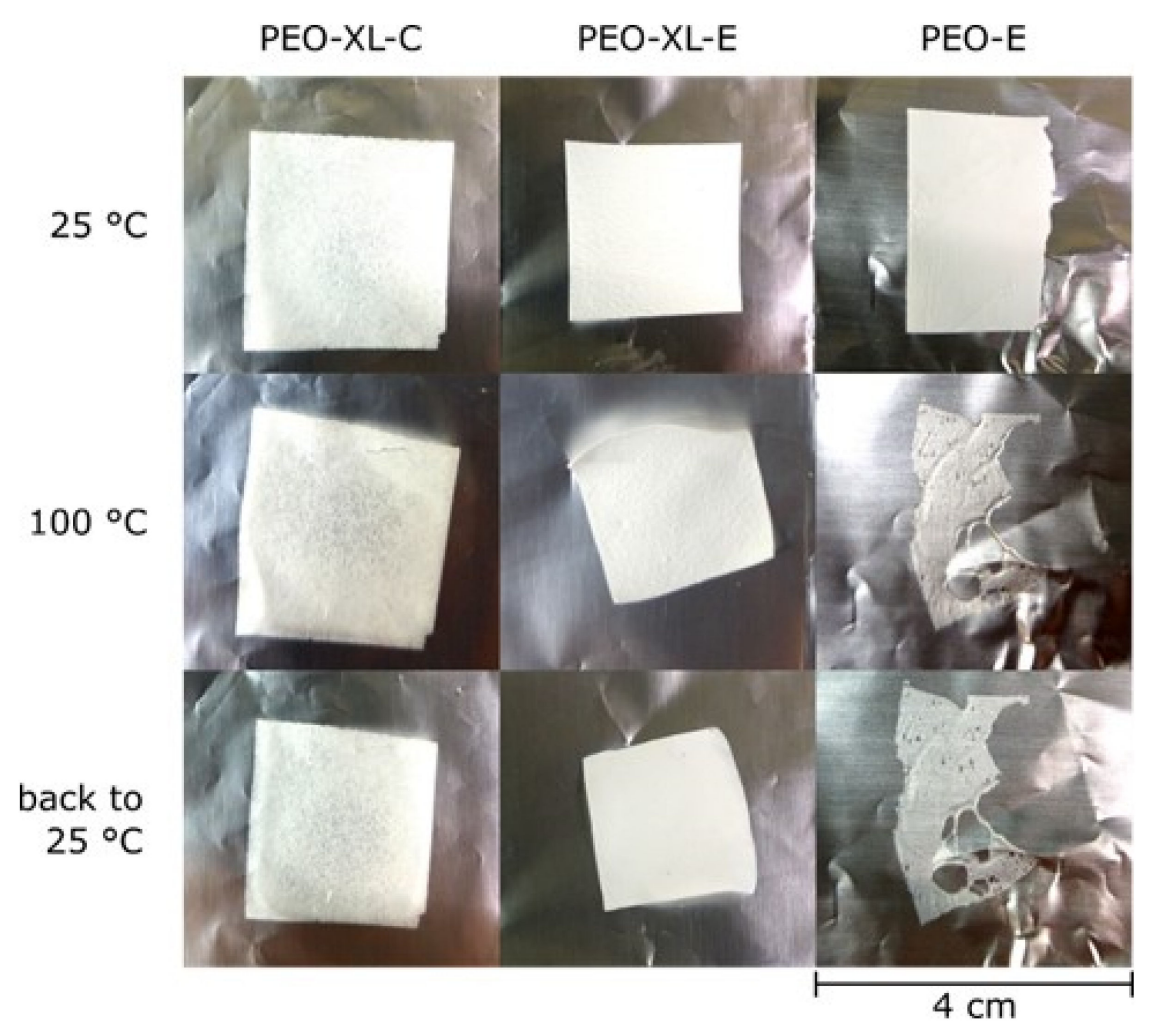
Pictures of the specimens at 25 °C (as prepared), at 100 °C for 2 min, and back to 25 °C.

However, although the crosslinking procedure decreases the total heat exchange, it is fundamental for producing a truly shape-stabilized PCM. This fact, already suggested by DMTA tests, is more clearly demonstrated in Figure 7, which shows specimens of the three prepared samples on a hot plate in laboratory conditions below (25 °C) and above (100 °C) the melting temperature of PEO. It can be observed that PEO-E completely loses the shape and melts on the aluminum substrate, while both efficiently crosslinked samples visually remain unchanged and keep their shape. The samples were also weighed prior to and after thermal treatment to investigate their potential leakage: PEO-XL-E and PEO-XL-C both presented no weight loss, thus being demonstrated to not lose melted PEO on the substrate even at high temperature.

**Figure 8:**
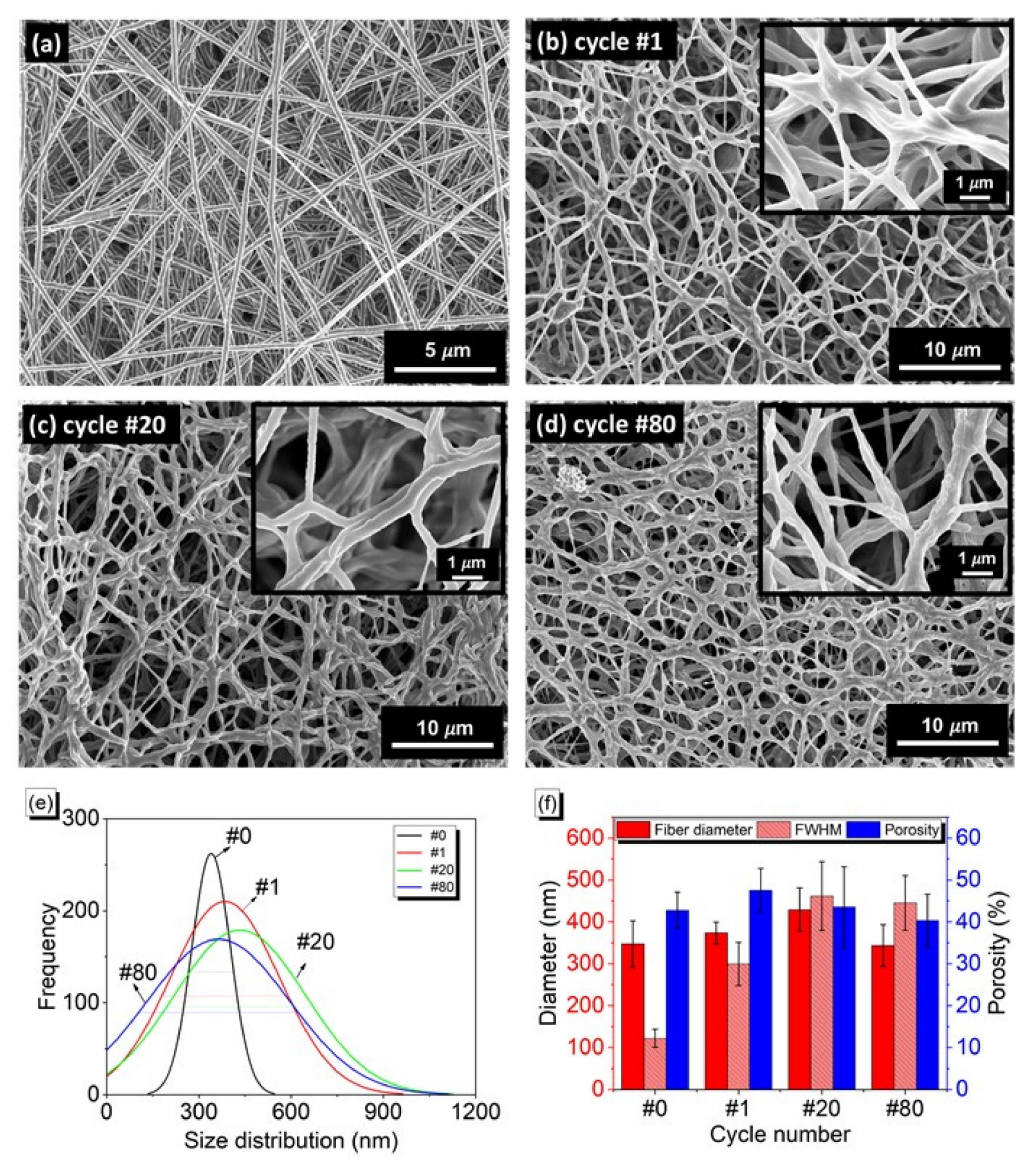
FE-SEM micrograph of PEO-XL-E as prepared (**a**) and after 1 (**b**), 20 (**c**), and 80 (**d**) thermal cycles in the climatic chamber; the insets are FE-SEM images in greater magnification. (**e**) size distribution of fibers before (#0) and after (#1, #20, and #80) thermal cycles. (**f**) fiber diameters, FWHM and surface porosity of the electrospun mats, obtained by DiamaterJ, after thermal cycling.

The characterization of the shape stability of the prepared fibrous mats was brought further: specimens of PEO-E and PEO-XL-E of 1 cm^2^ were put in a climatic chamber and cycled for 80 times between 10 °C and 100 °C. Samples were collected after 1, 20, and 80 cycles and analyzed with FE-SEM. The sample PEO-E loses the fibrous morphology already after the first cycle, while PEO-XL-E keeps the fibrous morphology even after 80 cycles (Figure 8).

With an increase in the number of cycles, as expected, the adjacent fibers weld together and undergo some morphology changes. The fibers diameter and surface porosity of the PEO-XL-E are characterized: as indicated in Figure 8e–f, the mean value of fiber diameters of PEO-XL-E does not considerably change after the cyclic test, while the FWHM of the size distribution reflects stronger changes particularly in samples subjected to 20 and 80 cycles. As prepared, PEO-XL-E samples show a narrow size distribution in a similar range of 150 nm to 550 nm with FWHM of 122 nm (Figure 2), whereas after 80 thermal cycles, a wider size distribution with ranges up to 1200 nm with FWHM of 460 nm were obtained. Additionally, the surface porosity does not exhibit significant changes with thermal cycling experiments (Figure 8f). Therefore, although the fibrous morphology goes through some distortion, these tests demonstrate that the crosslinking treatment is effective to preserve the specimen integrity above the melting temperature not only macroscopically, but also from the point of view of the micro- and nano-scopic fibrous structure.

## 4. Conclusions

PEO was electrospun in presence of a multifunctional acrylic crosslinker (trimethylolpropane triacrylate) and a photoinitiator (benzophenone), and the electrospun mats were subsequently subjected to UV-curing. The efficiently UV-cured PEO electrospun material showed remarkable water resistance, keeping its nanofibrous morphology after immersion in water, as verified through FE-SEM imaging. UV-crosslinking was also proven to effectively enhance the mechanical properties. In fact, the crosslinked mat showed a five-fold increase in both the tensile strength (from 0.15 MPa to 0.74 MPa) and strain at break (from 2.5% to 12.2%) compared to the uncrosslinked mat. Moreover, DMTA measurements showed that unlike uncured-PEO electrospun mat, the crosslinked system kept its integrity also above its melting temperature, with measurable values of storage and loss moduli. Such samples retained not only the macroscopic shape, remaining visually unchanged, but also the nanofibrous morphology, even after 80 thermal cycles, as proved by FE-SEM imaging. Thermal and heat storage/management properties of the PEO crosslinked electrospun mat evidenced a remarkable phase change enthalpy (≈112 J/g), which is kept stable over 50 DSC heating/cooling cycles.

These results evidence that UV-crosslinking is effective in stabilizing electrospun PEO nanofibers not only against dissolution in water, but also against melting, thus establishing the crosslinked electrospun PEO-based mats as potential shape-stabilized PCM in heat storage/management applications and in thermoregulating textiles.

## Figures and Tables

**Table 1:** List of prepared samples with nominal composition, processing methods, and obtained form.

Sample	Composition	Processing	Sample Form
PEO-E	PEO; BP	Electrospinning + UV irradiation	Fibrous mat
PEO-XL-E	PEO; BP; TMPTA	Electrospinning + UV irradiation	Fibrous mat
PEO-XL-C	PEO; BP; TMPTA	Casting + UV irradiation	Continuous film

**Table 2:** Results of C=C conversion and gel content measurement.

Sample	C=C Conversion (%)	Gel Content (%)
PEO-XL-E	44.6 ± 3.9	76.0 ± 3.3
PEO-XL-C	38.3 ± 5.2	95.8 ± 0.6

**Table 3:** Tg values obtained by DMTA measurements as the temperature corresponding to the maximum of the *E″* peak.

Sample	$T_{g}\;({}^{\circ}C)$
PEO-E	−17 ± 4
PEO-XL-E	−20 ± 1
PEO-XL-C	−28 ± 2

**Table 4:** Main results of DSC tests on the prepared samples.

Sample	$T_{m_{1}}$ (°C)	$\Delta H_{m_{1}}\;$ (J/g)	$T_{c}$ (°C)	$\Delta H_{c}$ (J/g)	$T_{m_{2}}$ (°C)	$\Delta H_{m_{2}}$ (J/g)
PEO-E	62.2	186.2	40.8	160.9	60.6	169.7
PEO-XL-E	63.8	161.2	41.1	102.5	58.8	112.3
PEO-XL-C	68.6	179.6	43.9	105.1	60.0	115.9

$T_{m_{1}}$ = melting temperature (first heating scan); $\Delta H_{m_{1}}$ = melting enthalpy (first heating scan); $T_{c}$ = crystallization temperature; $\Delta H_{c}$ = crystallization enthalpy; $T_{m_{2}}$ = melting temperature (second heating scan)—absolute minimum; $\Delta H_{m_{2}}$ = melting enthalpy (second heating scan).

## Data Availability

The data presented in this study are contained within the article or supplementary material, and are available on request from the corresponding authors.

## Supplementary Materials

The following are available online at https://www.mdpi.com/article/10.3390/polym13172979/s1, Figure S1: DMTA thermograms; Table S1: *E*″ peak temperature from DMTA analysis; Measurement of the temperature trend during thermal cycling; Figure S2. Results of the climatic chamber tests.
