# Supplementary material for: Electrospun Shape-Stabilized Phase Change Materials Based on Photo-Crosslinked Polyethylene Oxide
Source: Polymers (Basel). 2021 Sep 2;13(17):2979. doi: 10.3390/polym13172979 (PMC8434372; doi:10.3390/polym13172979)
Supplement: Supplementary file 1 [file polymers-13-02979-s001.zip › polymers-1348117-supplementary.pdf]

Supplementary Material

Electrospun shape-stabilized phase change materials based on photo-crosslinked polyethylene oxide

Giulia Fredi[a,b,#,*], Parnian Kianfar[c,#], Sara Dalle Vacche[c,d], Alessandro Pegoretti[a,b], and Alessandra Vitale[c,d,*]

[a] University of Trento, Department of Industrial Engineering, 38123 Trento, Italy

[b] INSTM – University of Trento Research Unit, 50121 Firenze, Italy

[c] Politecnico di Torino, Department of Applied Science and Technology, 10129 Torino, Italy

[d] INSTM – Politecnico di Torino Research Unit, 50121 Firenze, Italy

[#] These authors contributed equally to this work.

* Corresponding authors: giulia.fredi@unitn.it (GF); alessandra.vitale@polito.it (AV)

DMTA analyses

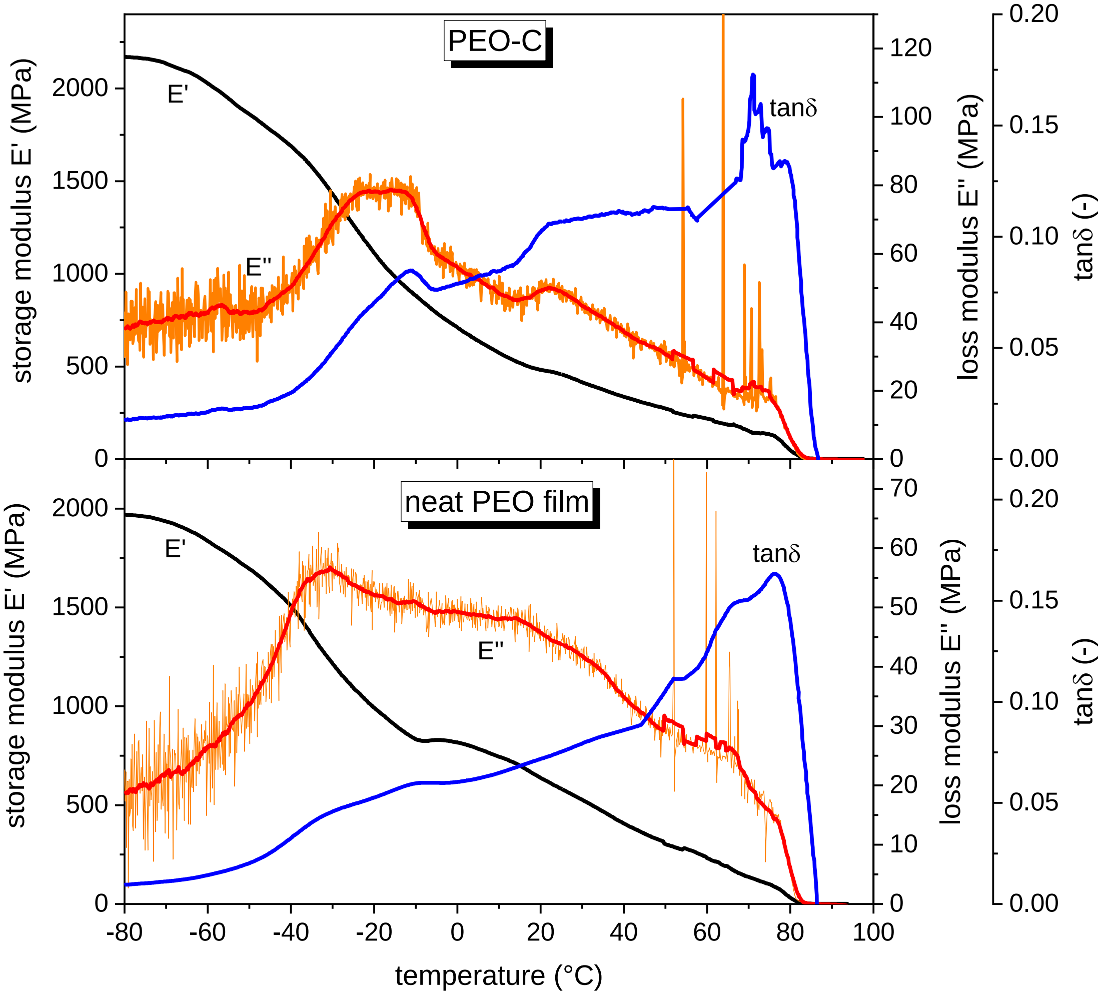

Figure S1. DMTA thermograms of the samples PEO-C (cast PEO film containing photoinitiator and subjected to UV-irradiation) and neat PEO cast film. To show the noisiness of the measurement data, E'' values have been reported both as acquired and after the smoothing process, necessary to identify a peak temperature.

Table S1. *E''* peak temperature, related to the glass transition temperature of PEO, for the tested samples, according to their composition and morphology.

	PEO+BP+TMPTA	PEO+PB	Neat PEO
Electrospun mat	-20 ± 1 [(a)]	- 17 ± 4 [(c)]	not tested
Cast film	38.3 ± 5.2 [(b)]	- 22 ± 2 [(d)]	-34 ± 1 [(e)]

BP = benzophenone (photoinitiator); TMPTA = trimethylolpropane triacrylate (crosslinker)
[(a)] *Sample PEO-XL-E*
[(b)] *Sample PEO-XL-C*
[(c)] *Sample PEO-E*
[(d)] *Sample PEO-C*
[(e)] *Neat PEO film*

Measurement of the temperature trend during thermal cycling

To have more information on the temperature trends during thermal cycling, PEO-XL-E samples were thermally cycled, and some key temperature values were measured with a RS-1384 data logging thermometer (RS Components S.r.l., Milano, Italy) equipped with four thermocouples. The first thermocouple (T_1) was wrapped with a specimen of fibrous membrane PEO-XL-E of approx. 5 cm^2, placed on an aluminum foil, while another thermocouple was wrapped with an empty aluminum foil of the same size as the first (T_2), used as reference sample (to simulate the thickness of PEO-XL-E, the aluminum foil was doubled in this case). Two additional thermocouples, T_3 and T_4, were placed freely in the climatic chamber and fixed near T_1 and T_2, respectively, to check the experimental temperature in the vicinity of the two specimens, as it may be slightly different in different zones of the climatic chamber. The temperatures T_3 and T_4 were in fact measured to sample the experimental temperature of the climatic chamber and to take into account small temperature variations of the in the climatic chamber, which has an internal volume of 340 liters.

Figure S2 shows the temperature trends of the four thermocouples. Due to the thin thickness of PEO-XL-E, it is very difficult to locate the thermocouple precisely and to appreciate a temperature plateau of T_1 around the phase change temperature of PEO (50-70 °C), but still the experiment was designed to demonstrate the advantage of using such material. In fact, **Figure S2** shows that, without PEO-XL-E, the maximum temperature difference ($T_4 - T_2$) is 2 °C on average, while with PEO-XL-E the maximum temperature difference ($T_3 - T_1$) is up to 6 °C, which indicates that a layer of PEO-XL-E helps to mitigate the temperature peaks.

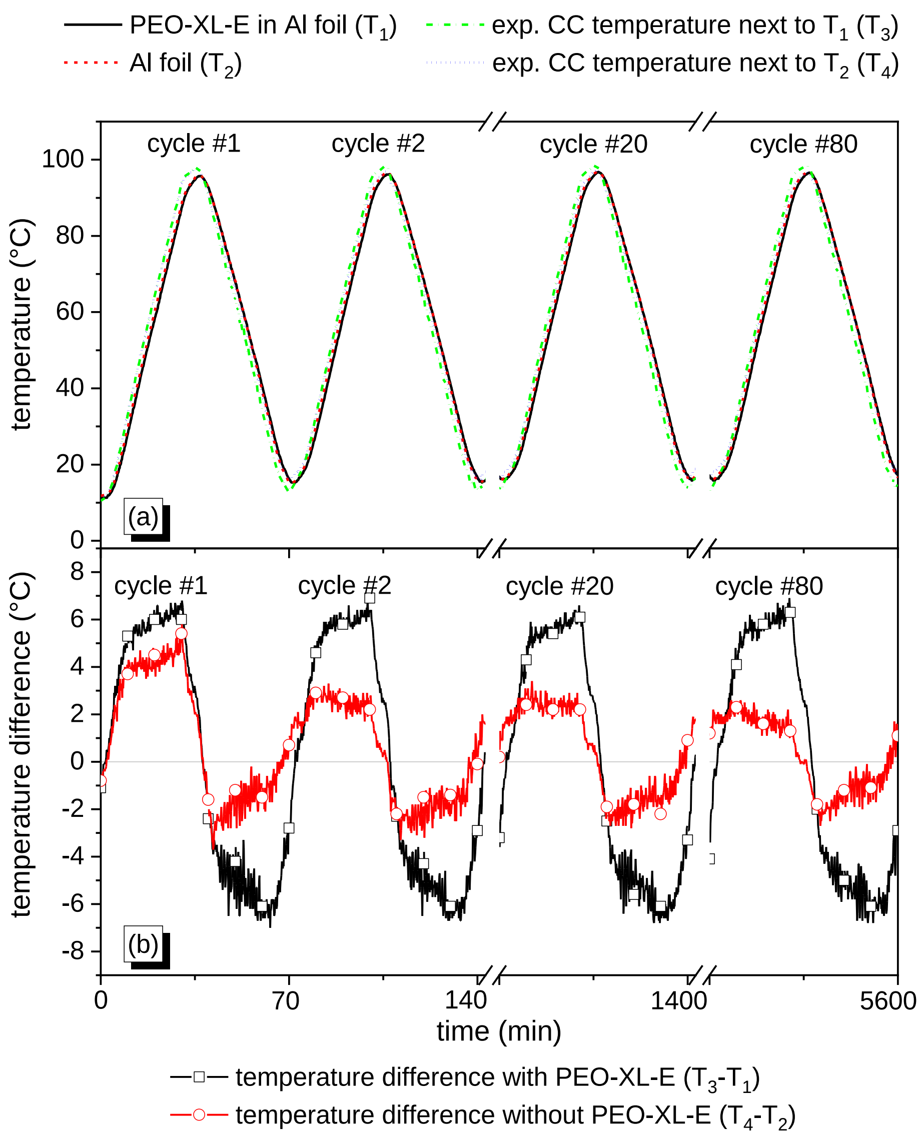

Figure S2. Results of the climatic chamber tests. (a) temperature trends; (b) temperature difference (CC = climatic chamber).
